# Doppler velocimetry of the orbital arteries in patients with sickle cell anemia: relationship with biomarkers of hemolysis

**DOI:** 10.1590/0100-3984.2015.0180

**Published:** 2017

**Authors:** Thiago de Oliveira Ferrão, Paulo Ricardo Martins-Filho, Cleverton Aragão, Marlyson Santana, Allan Nascimento, Thayane Cardoso, Rosana Cipolotti

**Affiliations:** 1 PhD, Radiologist, Assistant Professor in the Department of Medicine at the Universidade Federal de Sergipe (UFS), Aracaju, SE, Brazil; 2 PhD, Adjunct Professor in the Graduate Program in Health Sciences and Head of the Laboratory of Investigative Pathology at the Universidade Federal de Sergipe (UFS), Aracaju, SE, Brazil; 3 Medical Student at the Universidade Federal de Sergipe (UFS), Aracaju, SE, Brazil; 4 PhD, Pediatric Oncologist/Hematologist, Associate Professor in the Department of Medicine at the Universidade Federal de Sergipe (UFS), Aracaju, SE, Brazil

**Keywords:** Anemia, sickle cell, Ultrasonography, Doppler, Orbit/blood supply, Hemolysis, Biomarkers

## Abstract

**Objective:**

To investigate orbital vascular resistance by Doppler velocimetry in patients with steady-state sickle cell anemia, as well as to characterize its relationship with biomarkers of hemolysis.

**Materials and Methods:**

This was a cross-sectional study of two groups: 71 outpatients with sickle cell anemia; and 32 age- and gendermatched, healthy subjects (control group). All participants underwent Doppler velocimetry of the orbital arteries and laboratory tests.

**Results:**

All biochemical laboratory markers were abnormal in the sickle cell anemia patients (*p* < 0.0001 vs. controls). In the patient group, Doppler velocimetry revealed increased flow velocities in the ophthalmic artery and reduced flow velocities in the central retinal artery, as well as high values for the resistance index (RI) and pulsatility index (PI) in both arteries (*p* < 0.0001 vs. controls). Biomarkers of hemolysis were found to correlate significantly with the RI and PI indices. In the ophthalmic artery, the reticulocyte count showed a moderate direct correlation with RI and with PI. In the central retinal artery, hemoglobin showed a strong inverse correlation with RI and with PI.

**Conclusion:**

Orbital vascular resistance, as evaluated by Doppler velocimetry, is elevated in patients with steady-state sickle cell anemia and shows a significant correlation with biomarkers of hemolysis.

## Abstract (pt)

**Objetivo:**

Investigar o estado de resistência vascular arterial orbitária por meio de dopplervelocimetria em portadores de anemia falciforme e sua relação com biomarcadores de hemólise.

**Materiais e Métodos:**

Dois grupos foram examinados neste estudo transversal: um grupo composto de 71 pacientes portadores de anemia falciforme, e o outro grupo, controle, formado por 32 sujeitos saudáveis, pareados por gênero e idade. Todos os participantes foram avaliados por meio de ultrassonografia Doppler das órbitas e de testes laboratoriais.

**Resultados:**

Todos os marcadores laboratoriais foram anormais nos portadores de anemia falciforme em comparação aos controles (*p* < 0,0001). A avaliação dopplervelocimétrica mostrou velocidades aumentadas na artéria oftálmica e reduzidas na artéria central da retina, bem como índices de resistência (IR) e de pulsatilidade (IP) vascular elevados em ambas as artérias no grupo anemia falciforme em relação ao grupo controle (*p* < 0,0001). A análise da relação entre os marcadores de hemólise e os índices IR e IP mostrou associação significativa, com moderada correlação direta entre reticulócitos e IR e IP na artéria oftálmica e forte correlação inversa entre hemoglobina e IR e IP na artéria central da retina.

**Conclusão:**

A resistência vascular arterial das órbitas, aferida por meio de dopplervelocimetria, é elevada em portadores de anemia falciforme, com moderada a forte correlação com os biomarcadores de hemólise.

## INTRODUCTION

Sickle cell anemia, also known as homozygous sickle cell disease (HbSS), is the most common hereditary hemoglobinopathy^(1,2)^. Estimates suggest that 250,000 children across the globe are born annually with HbSS. Brought to the Americas through the slave trade, HbSS is most common in communities where individuals of African descent predominate. It is estimated that approximately 2,500 children are born annually with HbSS in Brazil. Non-whites were estimated at 50.74% of the population in the 2010 Census, and 1–6% of such individuals reportedly have HbSS^(3)^.

Sickle cell disease is characterized by the abnormal production of hemoglobin, also known as hemoglobin S. Sickle-shaped erythrocytes are formed during the polymerization and intracellular precipitation of hemoglobin S in its deoxygenated state, creating cellular rigidity that reduces the microvascular blood flow, leading to tissue ischemia and cardiac arrest^(4,5)^. Hemolysis also plays a significant role in the pathogenesis of the disease, possibly by reducing the bioavailability of the nitric oxide produced by vascular endothelial cells^(6)^. The clinical manifestations of this multisystemic disease stem from chronic hemolytic anemia and from the vaso-occlusive effects of the sickle cells, which affect nearly all tissues in the body, including those of the eyes.

The most significant ocular manifestations of sickle cell disease are in the retina, more frequently in patients with one of the double heterozygous forms of the disease (HbSC) than in those with HbSS^(7)^, and can be grouped into proliferative and nonproliferative forms. In the retina, various pathological processes may occur (hypoxia, ischemia, neovascularization, and fibrovascularization stemming from microvascular occlusion resulting from HbSS. The formation of new blood vessels is the most important factor that can lead to amaurotic complications that precede the occurrence of vitreous hemorrhage or retinal detachment. Asymptomatic ocular conditions may develop, regardless of the progression of the disease, and can have devastating consequences^(8)^.

Doppler ultrasound of the eye permits the noninvasive evaluation of arterial vascular resistance through the calculation of two indices-the resistance index (RI) and the pulsatility index (PI)-especially in the study of blood flow in ocular diseases^(9)^. Earlier studies have described Doppler velocimetry findings in intracranial, pulmonary, renal, and peripheral arteries in patients with HbSS^(4,10-14)^. To our knowledge, however, little is known about the orbital vascular changes in patients with HbSS, or about the relationships such changes have with biomarkers of hemolysis. The objective of this study was to investigate the state of arterial vascular resistance in and around the eye by Doppler velocimetry in patients with HbSS and to determine its relationship to biomarkers of hemolysis.

## MATERIALS AND METHODS

This was an analytical cross-sectional study conducted from September 2012 to October 2013 at in the Department of Diagnostic Imaging and the Pediatric Hematology/Oncology Outpatient Clinic at the University Hospital of the Federal University of Sergipe.

### Sample size

To calculate the sample size (n), we employed the following formula: *n* = (Z_α/2_ *σ/E)^2^, where: Z_α/2_ represents the critical value of Z for a confidence interval of 95%; σ is the standard deviation in the population; and E is the margin of error. Because the value of s is unknown, it was replaced with the standard deviation in the sample (s). Based on a preliminary pilot study involving 39 patients, the values *s* = 13.05 and *E* = 3.0 were calculated, whereas a value of Z_α/2_ = 1.96 was obtained from the statistics table, resulting in an estimated sample size of 73 patients.

Two groups (a total 105 subjects) were examined. One group composed of 73 patients with HbSS (confirmed by hemoglobin electrophoresis), of whom 38 were male. Ages ranged from 3 to 31 years. Those 73 patients were under regular follow-up treatment at an outpatient clinic, were in a compensated state, and had not had a severe pain episode or required a blood transfusion in the last three months. "Compensated" here means the patient is in a basal state without acute pain requiring hospitalization. The other group was composed of 32 apparently healthy controls, matched for age and gender, of whom 16 were male. Ages ranged from 3 to 27 years. The patients in the control group had no clinical history of pathological changes. The controls were recruited by random selection of patients undergoing routine ultrasound examination of unrelated organs and systems in the Department of Diagnostic Imaging. None of the patients in either group showed objective signs of ocular involvement.

Of the group of 73 patients with HbSS, 2 were excluded for lack of cooperation. None of the remaining participants were lost to follow-up. The final sample therefore consisted of 71 patients and 32 controls. The study was approved by the Research Ethics Committee of the University Hospital, and all participants gave written informed consent.

### Ultrasound protocol

The ultrasound evaluation was conducted with a Logiq P6 system (GE Healthcare; Milwaukee, WI, USA), which has color and pulsed Doppler capability, with a linear (11L), multifrequency (3.4–10.8 MHz) transducer, according to a standardized technique^(15,16)^. The patients were examined in the supine position, looking straight ahead but with their eyes closed. A drop of gel was applied to the transducer, which was then positioned transversely on the upper eyelid. The examination was performed under comfortable conditions with a non-compressive technique to avoid pressure from the transducer on the eyelid interfering with the results of the Doppler velocimetry. The ophthalmic and central arteries of the retina were identified with the aid of the color mode, which helped in the positioning of the sample volume, which was adjusted between 2 mm and 4 mm at the smallest possible angle of insonation, for the measurement of the Doppler velocimetry variables. The following Doppler velocimetry parameters were evaluated: peak systolic velocity (PSV), final diastolic velocity (FDV), mean velocity (MV), RI, and PI. Each parameter was measured only after at least three consecutive images of similar appearance had been observed in the spectral mode. Measurements were obtained with the resources available in the ultrasound system software. The RI and PI were calculated in a conventional way with the following formulas: *RI = (PSV – FDV)/PSV* and *PI = (PSV – FDV)/MV*. The average of three readings of each parameter for each artery in both orbits was obtained and recorded for each participant. The average duration of the ultrasound examination was 10–15 min per patient, depending on subject cooperation in keeping their eyes immobile. Heart rate and blood pressure were measured prior to the procedure. At the time of the examination, all of the participants had a normal heart rate and were normotensive. All examinations were performed by the same radiologist (with seven years of experience), who was blinded to the allocation of the subjects.

### Biochemical investigation

Blood samples were collected for complete blood counts, obtained with an automated analyzer (Labmax; Labtest, Minas Gerais, Brazil), and for erythrocyte counts, as well as for the determination of hemoglobin, hematocrit, mean corpuscular volume, mean corpuscular hemoglobin, mean corpuscular hemoglobin concentration, and the reticulocyte count. The biochemical investigation was carried out in an automated hematology analyzer (Cell-Dyn; Abbott Laboratories, Abbott Park, IL, USA), with the determination of the total and indirect levels of bilirubin, as well as that of the level of lactate dehydrogenase. As biomarkers of chronic intravascular hemolysis^(5)^, we considered hemoglobin levels, reticulocyte counts, lactate dehydrogenase levels, and (total and indirect) bilirubin levels.

### Statistical analysis

The distribution of patient groups and controls was tested for normality of data distribution (by the D'Agostino-Pearson test). The mean and standard deviation were calculated for age of the subject, biochemical variables and Doppler velocimetry variables (PSV, FDV, MV, RI, and PI). For the comparison of groups, the values of p were calculated by unpaired *t*-test or Mann-Whitney test. Multiple linear regression was used in order to determine the relationship between biomarkers of hemolysis and vascular resistance indices (RI and PI). Spearman's test was applied after the regression analysis to measure the correlation strength of the variables with statistical significance, as determined by calculating the linear correlation coefficient (*r*). The strength of the correlation was categorized, on the basis of the absolute value of r, as follows: 0 ≤ *r* < 0.2, very weak; 0.2 ≤ *r* < 0.4, weak; 0.4 ≤ *r* < 0.6, moderate; 0.6 ≤ *r* < 0.8, strong; 0.8 ≤ *r* < 1.0, very strong. All statistical tests were two-tailed, and values of *p* < 0.05 were considered significant. The statistical analysis was conducted using the BioStat program, version 5.3 (Analyst-Soft, Inc.; Alexandria, VA, USA).

## RESULTS

The groups did not differ in age or heart rate (Table 1). All biomarkers were abnormal in the patients with HbSS in comparison with the controls (*p* < 0.0001 for all comparisons).

**Table 1:** Clinical characteristics of patients with HbSS and healthy controls.

Characteristic	Patients			Controls		
	Mean	(SD)		Mean	(SD)	*p*-value
Age, years	14.3	(7.6)		16.4	(7.8)	0.21
HR, bpm	79.9	(13.2)		74.7	(11.7)	0.06
Hb, g/dL	7.69	(1.18)		13.68	(1.27)	< 0.0001
Ht, %	22.38	(3.55)		41.44	(3.30)	< 0.0001
Ec, ×106/µL	2.43	(0.37)		4.97	(0.43)	< 0.0001
Rc, %	11.82	(5.29)		0.94	(0.46)	< 0.0001
MCV, fL	91.17	(9.40)		76.75	(19.31)	< 0.0001
MCH, pg	31.32	(3.50)		34.44	(18.68)	< 0.0001
MCHC, g/dL	34.36	(1.57)		33.0	(1.36)	< 0.0001
LDH, U/L	1406.23	(544.29)		385.43	(129.93)	< 0.0001
TB, mg/dL	4.46	(3.47)		0.42	(0.19)	< 0.0001
IB, mg/dL	3.74	(3.19)		0.28	(0.15)	< 0.0001

HR, heart rate; Hb, hemoglobin; Ht, hematocrit; Ec, erythrocytes; Rc, reticulocytes; MCV, mean corpuscular volume; MCH, mean corpuscular hemoglobin; MCHC, mean corpuscular hemoglobin concentration; LDH, lactate dehydrogenase; TB, total bilirubin; IB, indirect bilirubin; SD, standard deviation.

Doppler velocimetry showed that, in the HbSS group, velocities were increased in the ophthalmic artery and reduced in the central retina artery, as well as that the RI and PI were elevated in both arteries (*p* < 0.0001 vs. the control group). The PSV of the central retinal artery was lower in the HbSS group, although the difference between the two groups was not statistically significant (Table 2). There were no significant differences between the left and right eyes of each participant, in terms of the Doppler velocimetry findings.

**Table 2:** Doppler velocimetry parameters for orbital vascular flows.

Parameters	Patients			Controls		
	Mean	(SD)		Mean	(SD)	*p*-value
Ophthalmic artery						
PSV, cm/s	32.9	(10.1)		24.0	(5.2)	< 0.0001
FDV, cm/s	10.3	(3.4)		8.4	(1.8)	0.0002
MV, cm/s	17.9	(5.4)		13.6	(2.9)	< 0.0001
RI	0.69	(0.05)		0.65	(0.03)	< 0.0001
PI	1.28	(0.19)		1.15	(0.09)	< 0.0001
Central retinal artery						
PSV, cm/s	10.3	(1.9)		11.0	(2.1)	0.070
FDV, cm/s	3.3	(0.8)		4.4	(0.9)	< 0.0001
MV, cm/s	5.6	(1.1)		6.6	(1.2)	0.0001
RI	0.68	(0.05)		0.59	(0.04)	< 0.0001
PI	1.26	(0.15)		1.00	(0.10)	< 0.0001

PSV, peak systolic velocity; FDV, final dyastolic velocity; MV, mean velociy; RI, resistance index; PI, pulsatility index; SD, standard deviation.

Multiple linear regression analysis of the relationship between the biomarkers of hemolysis and the indices of vascular resistance showed that the RI and PI correlated significantly with the reticulocyte count in the ophthalmic artery, as well as with the hemoglobin level in the central retinal artery (Tables 3 and 4, respectively). Neither lactate dehydrogenase nor bilirubin (total or indirect) correlated significantly with either index in any artery.

**Table 3:** Multiple linear regression of the effects of biomarkers of hemolysis on the RI in the ophthalmic and central retinal arteries.

Biomarker	Ophthalmic artery				Central retinal artery		
	R	T	*p*-value		R	T	*p*-value
Hb, g/dL	0	0.36	0.72		0	-5.52	< 0.0001
Rc, %	0	2.11	0.04		0	-1.14	0.26
LDH, U/L	0	-1.08	0.28		0	1.11	0.27
TB, mg/dL	0	0.47	0.64		0	1.67	0.1
IB, mg/dL	0	0.7	0.48		0	0.16	0.87

Hb, hemoglobin; Rc, reticulocytes; LDH, lactate dehydrogenase; TB, total bilirubin; IB, indirect bilirubin; R, multiple correlation coefficient; T, t statistical test.

**Table 4:** Multiple linear regression of the effects of biomarkers of hemolysis on the PI in the ophthalmic and central retinal arteries.

Biomarkers	Ophthalmic artery				Central retinal artery		
	R	T	*p*-value		R	T	*p*-value
Hb, g/dL	0	0.32	0.75		-0.04	-5.61	< 0.0001
Rc, %	0.01	2	0.05		0	-1.33	0.19
LDH, U/L	0	-1.21	0.23		0	1.03	0.31
TB, mg/dL	0	0.41	0.68		0.01	1.87	0.06
IB, mg/dL	0.1	0.62	0.53		0	0.12	0.9

Hb, hemoglobin; Rc, reticulocytes; LDH, lactate dehydrogenase; TB, total bilirubin; IB, indirect bilirubin; R, multiple correlation coefficient; T, t statistical test.

**Figure 1:**
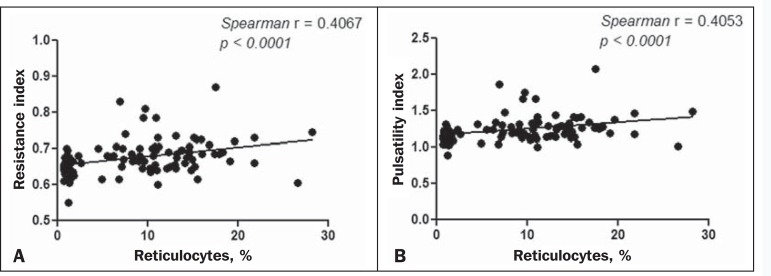
Correlation analysis between reticulocyte counts and the vascular indices (RI and PI) in the ophthalmic artery.

**Figure 2:**
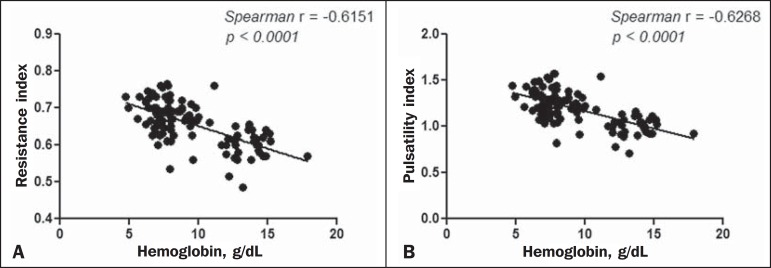
Correlation analysis between hemoglobin levels and the vascular indices (RI and PI) in the central retinal artery.

Because the multivariate analysis showed the RI and PI to correlate significantly with the reticulocyte count and hemoglobin level, we calculated the value of *r*, generating dispersion diagrams with an adjustment line for the bivariate analysis in order to illustrate and quantify this relationship in each artery. As can be seen in Figure 1, both indices presented a moderate direct correlation with the reticulocyte count in the ophthalmic artery (*r* = 0.41). Figure 2 shows that, in the central retinal artery, there was a strong inverse correlation between the hemoglobin level and RI (*r* = 0.62), as well as between the hemoglobin level and PI (*r* = 0.63).

## DISCUSSION

The ophthalmologic complications of HbSS can affect the orbit, conjunctiva, anterior chamber, and posterior segment. In the orbit, it manifests as orbital compression syndrome, caused by infarction of the sphenoid bone with subperiosteal hematoma formation and an inflammatory response. In the conjunctiva, vascular segments undergo transient saccular dilation, with a "sausage" or "comma" aspect, notably in the lower bulbar portion; the "comma sign" is more common in HbSS than in HbSC. Endothelial proliferation and aggregation of erythrocytes in the distal portion of the capillaries, together with dilation and thinning of proximal vessel segments, are the predominant histopathological findings; low levels of hemoglobin and hematocrit predispose to conjunctival changes^(17)^. Filled with aqueous humor, the anterior chamber is an environment with low pH and low oxygen concentration. In the setting of hemorrhage, erythrocytes and leukocytes consume oxygen and liberate carbonic gas and lactate, thus causing acidosis and sickling. The sickle cells obstruct the trabecular meshwork (responsible for the drainage of the aqueous humor), leading to an increase in intraocular pressure, also known as acute glaucoma, which constitutes an ophthalmologic emergency. In the posterior segment, manifestations include retinal hemorrhage and exudate, as well as angioid streaks, chorioretinal infarction, vitreous hemorrhage, occlusion of the central retinal artery or its branches, and proliferative retinopathy. Arteriolar occlusion and loss of capillary perfusion in the periphery of the retina are the most striking features of sickle cell retinopathy, being more common in HbSC hemoglobinopathy than in the HbSS form and occurring predominantly in the superior temporal region; ischemic areas release substances that stimulate angiogenesis, and the initial vascular remodeling at the junction between the nonperfused periphery and the perfused central region promotes the formation of arteriovenous anastomoses^(8,17)^.

Our data show increased velocities in the ophthalmic artery and reduced velocities in the central retinal artery, in agreement with the findings of Aikimbaev et al.^(15)^. We observed high resistance to flow in both vessels. However, Aikimbaev et al.^(15)^ detected increased RI and PI only in the central retinal artery, without significant changes in the ophthalmic artery. Tantawy et al.^(8)^ found that ophthalmic artery velocities were higher in children and adolescents with sickle cell disease than in healthy controls, although the difference was not statistically significant. The authors followed a protocol involving the use of transcranial Doppler ultrasound, with a standard 2.0-MHz transducer, which provides lower spatial resolution than does the 10.8-MHz probe used in the present study. The higher resolution facilitates the study of superficial structures, with greater sharpness and sensitivity in the Doppler velocimetry evaluation. That may have contributed to the detection of statistical significance in our sample.

Polska et al.^(18)^ stated that the retinal artery RI, as measured by Doppler ultrasound, does not correspond to true retinal vascular resistance, when measured by a much more complex method (laser Doppler velocimetry, vessel diameter measurements by a Zeiss analyzer with video camera and specific software, or measurement of ocular perfusion pressure by Goldmann tonometry). The authors found that, after the use of 100% oxygen as a retinal vasoconstrictor agent, the RI and the true resistance both demonstrated an increase in vascular resistance, although the effect detected by RI was lower. Those authors used transducers with a lower frequency than that of those employed in the present study (5.0 MHz vs. 10.8 MHz), and the software accompanying our equipment was more advanced than was that available 13 years ago, when Polska et al.^(18)^ published their data. Technological advances have allowed the improvement of diagnostic imaging tools in all areas, including Doppler ultrasound, with an increase in the accuracy of the measurements. Calculation of the RI currently allows better estimation of retinal vascular resistance than in the past decade. In addition, its use is more practical, available, and reproducible than the complex methodology proposed by Polska et al.^(18)^, as well as being much more affordable and not compromising the clinical evaluation of the patient.

The mean diameter of the ophthalmic artery is 1430 ± 260 µm^(19)^, compared with 163 ± 17 µm for the central retinal artery^(20)^; that is, the diameter of the central retinal artery is approximately ten times smaller than is that of the ophthalmic artery. The expected effect of vasoconstriction on the ophthalmic artery is increased flow velocity, as occurs in other arteries during pathological processes that promote luminal narrowing, such as atheromatous plaque stenosis in the internal carotid, renal, and femoral arteries. In contrast, the effect of vasoconstriction on an artery of very small diameter, such as the central retinal artery, could promote a reduction in flow velocity, because vasoconstriction of an already anatomically narrow lumen would have what amounts to a subocclusive effect. According to Poiseuille's Law, the volume of flow in a vessel is directly proportional to the fourth power of the radius of that vessel. The central retinal artery has a radius ten times smaller than that of the ophthalmic artery and would therefore have a flow volume ten thousand times lower. Any vasoconstrictive event, however mild, would have a hemodynamically significant repercussion, which would explain the finding of reduced velocity. Similar behavior is observed in the small vessels of the bulbar conjunctiva, when evaluated by computer-assisted intravital microscopy, a method used in order to characterize the microcirculation. In the presence of vasoconstriction, flow velocity decreases instead of increases^(21)^.

Regarding biomarkers of hemolysis, our study revealed a strong inverse correlation between the hemoglobin level and RI in the central retinal artery, and we found that the RI and PI showed a moderate direct correlation with the reticulocyte count in the ophthalmic artery. Aikimbaev et al.^(15)^ demonstrated a strong association between reduced levels of hemoglobin and increased retinal vascular resistance (relative risk: 6.7; *p* < 0.009), although we did not identify a statistically significant relationship between such resistance and reticulocyte counts. However, Cheung et al.^(21)^ did not find the hemoglobin level or the reticulocyte count to correlate significantly with the mean cerebral artery flow velocities, as measured by transcranial Doppler ultrasound, or with the velocities in the microcirculation of the bulbar conjunctiva, as evaluated by computer-assisted intravital microscopy. Neither of the studies cited above evaluated the other two biomarkers of hemolysis (lactate dehydrogenase and bilirubin).

Recent studies have classified the clinical complications of HbSS into two distinct subphenotypes^(5,22,23)^: vasculopathy/endothelial dysfunction/hemolysis (which includes pulmonary hypertension, priapism, lower limb ulcer, and stroke, more common in patients with higher rates of intravascular hemolysis); and viscosity/vaso-occlusion (encompassing painful vaso-occlusive crisis, acute thoracic syndrome, and osteonecrosis). The rate of chronic intravascular hemolysis can be estimated by identifying biomarkers, which are characterized as follows^(1,5)^: low hemoglobin levels; high lactate dehydrogenase levels; high bilirubin levels; and high reticulocyte counts. The literature does not mention the subphenotype to which sickle cell retinopathy belongs. Some evidence points to the first subphenotype: vascular fibroproliferation, which occurs both in proliferative retinopathy and in pulmonary hypertension-involving nitric oxide depletion and a metabolic shift from arginine and nitric oxide synthesis to proline and collagen synthesis^(8,17,22)^-and correlation with the biomarkers of hemolysis characterized in the present study (hemoglobin levels and reticulocyte counts). The vaso-occlusive manifestations in the conjunctiva bulbar and retina, however, suggest the second subphenotype^(17,21)^.Further studies, including molecular studies, are needed in order to improve our understanding of these subphenotypes.

To our knowledge, there have been no studies explaining why RI and PI correlate only with some biomarkers of hemolysis. This is an interesting starting point for additional investigations. Our study has certain limitations. For example, having a second radiologist would have allowed us to evaluate interobserver agreement through determination of the kappa index. In addition, for lack of an appropriate diagnostic tool, we did not study the retinal microcirculation.

## CONCLUSION

The present study demonstrated that arterial vascular resistance in the orbital arteries, as measured by Doppler ultrasound, is high in patients with HbSS, showing moderate to strong correlations with some biomarkers of hemolysis.
